# On the reconstruction of the ancestral bacterial genomes in genus *Mycobacterium* and *Brucella*

**DOI:** 10.1186/s12918-018-0618-2

**Published:** 2018-11-20

**Authors:** Christophe Guyeux, Bashar Al-Nuaimi, Bassam AlKindy, Jean-François Couchot, Michel Salomon

**Affiliations:** 1 FEMTO-ST Institute, UMR 6174 CNRS, DISC Computer Science Department, Univ. Bourgogne Franche-Comté (UBFC), 16 Route de Gray, Besançon, 25000 France; 2 Department of Computer Science, Al-Mustansiriyah University, Baghdad, 10052 Iraq; 3 Department of Computer Science, Diyala University, Diyala, 32001 Iraq

**Keywords:** Mycobacterium tuberculosis, Genome rearrangements, Ancestral reconstruction, Bacterial lineages, Pathogens, Evolution

## Abstract

**Background:**

To reconstruct the evolution history of DNA sequences, novel models of increasing complexity regarding the number of free parameters taken into account in the sequence evolution, as well as faster and more accurate algorithms, and statistical and computational methods, are needed. More particularly, as the principal forces that have led to major structural changes are genome rearrangements (such as translocations, fusions, and so on), understanding their underlying mechanisms, among other things via the ancestral genome reconstruction, are essential. In this problem, since finding the ancestral genomes that minimize the number of rearrangements in a phylogenetic tree is known to be NP-hard for three or more genomes, heuristics are commonly chosen to obtain approximations of the exact solution. The aim of this work is to show that another path is possible.

**Results:**

Various algorithms and software already deal with the difficult nature of the problem of reconstruction of the ancestral genome, but they do not function with precision, in particular when indels or single nucleotide polymorphisms fall into repeated sequences. In this article, and despite the theoretical NP-hardness of the ancestral reconstruction problem, we show that an exact solution can be found in practice in various cases, encompassing organelles and some bacteria. A practical example proves that an accurate reconstruction, which also allows to highlight homoplasic events, can be obtained. This is illustrated by the reconstruction of ancestral genomes of two bacterial pathogens, belonging in *Mycobacterium* and *Brucella* genera.

**Conclusions:**

By putting together automatically reconstructed ancestral regions with handmade ones for problematic cases, we show that an accurate reconstruction of ancestors of the *Brucella* genus and of the *Mycobacterium tuberculosis* complex is possible. By doing so, we are able to investigate the evolutionary history of each pathogen by computing their common ancestors. They can be investigated extensively, by studying the gene content evolution over time, the resistance acquisition, and the impacts of mobile elements on genome plasticity.

## Background

*Mycobacterium tuberculosis* (MTB) is the etiologic agent of human tuberculosis (TB), that is one of the oldest recorded human afflictions which is still among the main worldwide death causes. In 2015, more than 10 million people became ill with TB and approximately 2 millions died from the disease, almost exclusively in low and middle income countries. Moreover, it induces a major global health problem, since about one-third of the world’s population has latent TB. Hence this is the first infectious disease declared by the World Health Organization (WHO) as a global emergency. More precisely, tuberculosis is caused by pathogens belonging to the *Mycobacterium tuberculosis complex* (MTBC) which consists of different species that are typical human pathogens (*Micobacterium canettii*, *africanum*, and *tuberculosis*), rodent ones (*M. microti*), or even *Mycobacteria* with a large host spectrum like *bovis* [1, 2]. Even if these organisms are genetically similar, they exhibit large differences with regard to epidemiology, pathogenicity, and host spectrum. *Mycobacterium tuberculosis* spreads throughout the human population since thousands of years, as the TB form that attacks bone and causes skeletal deformities can be still identified on individuals who died from it several thousands years ago, like ancient Egyptian mummies with apparent tubercular deformities.

The MTBC species are classified in 6 phylogenetic lineages which can be further divided into sublineages showing phenotypic differences reflecting for example their virulence (pathogenicity). The species members of the *Mycobacterium tuberculosis complex* have a clonal structure with large genome similarity (more than 99.9 percent of DNA sequences in common [3]). Compared to more ancient species, this complex has more virulent chromosomes [4]. As they have the same ancestor [5], the fact that we can find rodent and human pathogens, and other with a larger spectrum, is indeed surprising. To study *M. tuberculosis* DNA sequence, its virulent laboratory strain *M. tuberculosis H37Rv* is commonly used. This strain consists of a single circular chromosome composed by 4,411,532 nucleotides and 3906 protein genes. DNA homology studies and comparison of 16S rRNA coding regions have permitted to establish how they are linked, showing a 95−100*%* DNA relatedness. For example, there is only one difference between the 16S rRNA gene sequence of *M. tuberculosis* and the one of *M. bovis*.

The long-term coevolution of *Mycobacterium tuberculosis* with humans [6] has led to a more or less large geographic spread of the different phylogenetic lineages of MTBC. Moreover, some of the lineages appear to have a large geographic distribution, while others seem to be restricted to a smaller group of human host populations. Over time, MTBC genomes have evolved through genomic repetition or replacement (insertion sequences, etc.) and genomic modification at different scales of complexity. In this latter case, modifications range from small-scale ones resulting from mutation or indels to larger ones occurring on DNA strands (inversion, duplication, or deletion).

Obviously, understanding the past and future evolution of the MTBC would be of great interest, leading to the ability to study the ancestors and to understand the evolution history of species, and finally to an improved knowledge of the mechanisms of resistance and virulence acquisition in human tuberculosis. Fortunately, the relatively short time-frame during which the MTBC emerged (this bacteria is quite recent [7]), the relatively low genomes lengths and the recombination scarcity, together with an easier access to ancient and current DNA sequences, are favourable factors to address this question. Therefore, it should be possible to design a model of evolution for this set of genomes, in order to recover their evolution history and to predict their future evolution.

Another interesting group of pathogenic bacteria to be investigated is the genus *Brucella* which causes *Brucellosis*, a disease that primarily affects animals, especially domesticated livestock, producing abortion and other reproductive disorders. Human can also be infected, mainly through animal-to-person spread, in which case long-lasting flu-like symptoms are observed. Like tuberculosis, brucellosis is a global problem, since it is the most common bacterial infection spread from animals to humans worldwide. After the recent identification of the species *B. vulpis*, a total of eleven species have been identified within the genus *Brucella* according to their pathogenicity and preferential animal host [8, 9], among which the six classically recognized species are: *B. melitensis, B. abortus, B. suis, B. ovis, B. canis*, and *B. neotomae*. *B. abortus* and *B. melitensis* are the most important species regarding prevalence and morbidity in humans and domestic animals.

Clearly, a detailed knowledge of the *Brucella* phylogeny would also be of great interest. First, the phylogenetic reconstruction can lead to an enhanced understanding of the ecology, evolutionary history, and host relationships of this genus. Second, it can be used to discover suitable genotyping methods for rapid detection and diagnostic measures, used for example in epidemiological studies to facilitate human disease research. Moreover, as the Brucella genus is highly conserved and has low genetic variation, the phylogenetic reconstruction is still a challenge, even if the *Brucella* genus is probably easier to tackle than the MTBC.

This requires the development of new algorithms for the detection and evolution of genomic changes. Researchers studying this question focus mainly on the nucleotidic mutations prediction, and take specific forms for the matrix of mutations that seem not in accordance with recent experimental evaluations, see [10]. These evolutionary models must be constructed in a different manner, to better reflect what really occurred. Moreover, the important effects of other genome changes (such as nucleotide insertions and deletions, large-scale recombination, or repeated sequence changes) have to be considered more deeply, and an effective ancestral reconstruction of ancient bacteria should be carried out.

This research work is an extension of an article presented to the 5th International Work-Conference on Bioinformatics and Biomedical Engineering (IWBBIO 2017, [11]). Its main objective is to show that, if we focus on strongly related bacterial chromosomes, the reconstruction of their most recent common ancestors is possible in practice. In order to do so, we propose a pragmatic approach that mix already published reconstruction algorithms with new original scripts and a human cross-validation. As an illustrative example, we provide the ancestral reconstruction of 65 genomes of the *Mycobacterium tuberculosis* complex, and of the 47 *Brucella* genomes that are available on the NCBI database.

The dynamics of the evolution process in DNA sequences results from local evolutionary events that consist in SNPs or indels. Genomic rearrangements, which are larger alterations of the genetic organization, can take the form of inversions and transpositions, or occur by chromosome fusion and fission. Obviously, over time such large-scale mutations have affected gene order and content, therefore they have a prominent role in speciation [12]. A key problem when studying evolutionary change at the level of a DNA sequence, which is investigated by the research work presented in this article, is the problem of ancestral sequence reconstruction. This one is as follows: given an evolutionary tree relating organisms and the DNA genomic sequences of the leaf species, predict the DNA sequence of all ancestral species in the tree. Many biological studies have addressed this problem and thus various methods have been proposed for inferring ancestral sequences. Apart from ancestral genome reconstruction problem, biomolecular evolution is usually devised through the evolution of core and pan-genome. Below is a brief overview on ancestral genome reconstruction.

Similarities in sequences or in the gene order (genome composition) are usually considered in up-to-date ancestral reconstruction methods. The first case, based on sequence similarity, can be considered as resolved now, at least when indels are not considered [13–21]. Indeed, considering a phylogenetic tree and its associated DNA alignment, Bayesian inference or maximum likelihood approaches can be applied to estimate ancestral states of nucleotides [22, 23]. The main problem is the insertion-deletion case, which is usually disregarded [24]. The small number of models that consider indels focus on the parcimony approach, or consider the evolutionary model called Thorne-Kishino-Felsenstein [25]. Combinatorics investigations are applied in the case of larger modifications, by modeling these recombinations as permutations of homologous sequences. This reformulation leads to the well-known genome rearrangement problem [26], in which the shortest edit operations that can map one chromosome to another are searched. Note that this NP-hard problem [12, 27] is directly related to the sequence length and the number of mutations, while genomes considered in this article are quite small and have faced only a low amount of recombination: the difficulty can be circumvented for such genomes.

The remainder of this article is organized as follows. The methodology proposed for ancestral reconstruction is detailed in the next section. Results of the application of this approach on the *Mycobacterium tuberculosis* complex (specifically on two of its species, namely *M. tuberculosis* and *M. canettii*) and on the *Brucella* genus case (focusing specifically on the *B. abortus* and *B. melitensis* species) are investigated in the third section. Finally, this research article ends with a discussion and a conclusion with future work.

## Methods

Let us now detail our concrete ancestral reconstruction for bacterial genomes, illustrated through a first set of strains detailed hereafter.

### Data acquisition and processing

A python script has firstly been written to automatically download all the complete genomes of *Mycobacterium* genus available on the NCBI database, encompassing 2 *africanum*, 15 *bovis*, 5 *canettii*, 1 *microti*, and 42 *tuberculosis*. Note that *canettii* and *tuberculosis* are well represented in this dataset, which is helpful to study how virulence has appeared in the first species, and if the second one is at the origin of the MTBC complex 40,000 years ago. Details about these 65 genomes are provided in Table 1.

**Table 1:** The considered *Mycobacterium* strains

Accession (GenBank)	Organism name	Sequence length (bp)	Nickname
CP010335.1	*Mycobacterium tuberculosis strain 2242*	4,419,839	*tuberculosis1*
CP010336.1	*Mycobacterium tuberculosis strain 2279*	4,405,033	*tuberculosis2*
NC_000962.3	*Mycobacterium tuberculosis H37Rv*	4,411,532	*tuberculosis3*
NC_002755.2	*Mycobacterium tuberculosis CDC1551*	4,403,837	*tuberculosis4*
NC_009525.1	*Mycobacterium tuberculosis H37Ra*	4,419,977	*tuberculosis5*
NC_009565.1	*Mycobacterium tuberculosis F11*	4,424,435	*tuberculosis6*
NC_012943.1	*Mycobacterium tuberculosis KZN 1435*	4,398,250	*tuberculosis7*
NC_016768.1	*Mycobacterium tuberculosis KZN 4207*	4,394,985	*tuberculosis8*
NC_016934.1	*Mycobacterium tuberculosis UT205*	4,418,088	*tuberculosis9*
NC_017522.1	*Mycobacterium tuberculosis CCDC5180*	4,405,981	*tuberculosis10*
NC_017524.1	*Mycobacterium tuberculosis CTRI-2*	4,398,525	*tuberculosis11*
NC_018078.1	*Mycobacterium tuberculosis KZN 605*	4,399,120	*tuberculosis12*
NC_018143.2	*Mycobacterium tuberculosis H37Rv*	4,411,709	*tuberculosis13*
NC_020089.1	*Mycobacterium tuberculosis 7199-99*	4,421,197	*tuberculosis14*
NC_020559.1	*Mycobacterium tuberculosis str. Erdman = ATCC 35801 DNA*	4,392,353	*tuberculosis15*
NC_021054.1	*Mycobacterium tuberculosis str. Beijing/NITR203*	4,411,128	*tuberculosis16*
NC_021194.1	*Mycobacterium tuberculosis EAI5/NITR206*	4,390,306	*tuberculosis17*
NC_021251.1	*Mycobacterium tuberculosis CCDC5079*	4,414,325	*tuberculosis18*
NC_021740.1	*Mycobacterium tuberculosis EAI5*	4,391,174	*tuberculosis19*
NC_022350.1	*Mycobacterium tuberculosis str*	4,408,224	*tuberculosis20*
NZ_AP014573.1	*Mycobacterium tuberculosis str. Kurono DNA*	4,415,078	*tuberculosis21*
NZ_CP002871.1	*Mycobacterium tuberculosis HKBS1*	4,407,929	*tuberculosis22*
NZ_CP002882.1	*Mycobacterium tuberculosis BT2*	4,401,899	*tuberculosis23*
NZ_CP002883.1	*Mycobacterium tuberculosis BT1*	4,399,405	*tuberculosis24*
NZ_CP002885.1	*Mycobacterium tuberculosis CCDC5180*	4,414,346	*tuberculosis25*
NZ_CP007027.1	*Mycobacterium tuberculosis H37RvSiena*	4,410,911	*tuberculosis26*
NZ_CP007803.1	*Mycobacterium tuberculosis K*	4,385,518	*tuberculosis27*
NZ_CP007809.1	*Mycobacterium tuberculosis strain KIT87190*	4,410,788	*tuberculosis28*
NZ_CP009100.1	*Mycobacterium tuberculosis strain ZMC13-264*	4,411,507	*tuberculosis29*
NZ_CP009101.1	*Mycobacterium tuberculosis strain ZMC13-88*	4,411,515	*tuberculosis30*
NZ_CP009426.1	*Mycobacterium tuberculosis strain 96075*	4,379,376	*tuberculosis31*
NZ_CP009427.1	*Mycobacterium tuberculosis strain 96121*	4,410,945	*tuberculosis32*
NZ_CP009480.1	*Mycobacterium tuberculosis H37Rv*	4,396,119	*tuberculosis33*
NZ_CP010330.1	*Mycobacterium tuberculosis strain F28*	4,421,903	*tuberculosis34*
NZ_CP010337.1	*Mycobacterium tuberculosis strain 22115*	4,401,829	*tuberculosis35*
NZ_CP010338.1	*Mycobacterium tuberculosis strain 37004*	4,417,090	*tuberculosis36*
NZ_CP010339.1	*Mycobacterium tuberculosis strain 22103*	4,399,422	*tuberculosis37*
CP010340.1	*Mycobacterium tuberculosis strain 26105*	4,426,489	*tuberculosis38*
NZ_CP012090.1	*Mycobacterium tuberculosis W-148*	4,418,548	*tuberculosis39*
NZ_CP012506.1	*Mycobacterium tuberculosis strain SCAID 187.0*	4,379,515	*tuberculosis40*
NZ_HG813240.1	*Mycobacterium tuberculosis 49-02*	4,412,379	*tuberculosis41*
CP010329.1	*Mycobacterium tuberculosis strain F1*	4,428,621	*tuberculosis42*
NC_015758.1	*Mycobacterium africanum GM041182*	4,389,314	*africanum1*
CP010334.1	*Mycobacterium africanum strain 25*	4,386,422	*africanum0*
CP010333.1	*Mycobacterium microti strain 12*	4,370,115	*microti*
NC_015848.1	*Mycobacterium canettii CIPT 140010059*	4,482,059	*canettii0*
NC_019951.1	*Mycobacterium canettii CIPT 140070010*	4,525,948	*canettii1*
NC_019950.1	*Mycobacterium canettii CIPT 140060008*	4,432,426	*canettii2*
NC_019952.1	*Mycobacterium canettii CIPT 140070017*	4,524,466	*canettii3*
NC_019965.1	*Mycobacterium canettii CIPT 140070008*	4,420,197	*canettii4*
NC_002945.3	*Mycobacterium bovis AF2122/97*	4,345,492	*bovis0*
NC_008769.1	*Mycobacterium bovis BCG Pasteur 1173P2*	4,374,522	*bovis1*
NC_012207.1	*Mycobacterium bovis BCG str. Tokyo 172 DNA*	4,371,711	*bovis2*
NZ_CP003494.1	*Mycobacterium bovis BCG str. ATCC 35743*	4,334,064	*bovis3*
NC_016804.1	*Mycobacterium bovis BCG str. Mexico*	4,350,386	*bovis4*
NC_020245.2	*Mycobacterium bovis BCG str. Korea 1168P*	4,376,711	*bovis5*
NZ_CP009449.1	*Mycobacterium bovis strain ATCC BAA-935*	4,358,088	*bovis6*
NZ_AM412059.1	*Mycobacterium bovis BCG str. Moreau RDJ*	4,340,116	*bovis7*
NZ_CP008744.1	*Mycobacterium bovis BCG strain 3281*	4,410,431	*bovis8*
NZ_CP012095.1	*Mycobacterium bovis strain 1595*	4,351,712	*bovis9*
NZ_CP009243.1	*Mycobacterium bovis BCG strain Russia 368*	4,370,138	*bovis10*
NZ_CP013741.1	*Mycobacterium bovis strain BCG-1 (Russia)*	4,370,705	*bovis11*
CP010331.1	*Mycobacterium bovis BCG strain 26*	4,351,313	*bovis12*
CP010332.1	*Mycobacterium bovis strain 30*	4,336,227	*bovis13*
NZ_CP014566.1	*Mycobacterium bovis BCG str. Tokyo 172 substrain TRCS*	4,371,707	*bovis14*

After the data acquisition stage, the next step is to align the downloaded sequences [28, 29]. Prior to the Multiple Sequence Alignment (MSA), genomes must be operated such that each sequence starts to the same location and is read in the same direction: we deal with circular genomes. This is why a sequence of reference (200 bp from *M. tuberculosis H37Rv*) and its reverse complement have been blasted locally. Then, a circular shift and/or a reverse complement of the whole sequence have been applied when required.

Most of the well-known alignment tools have failed to align these genomes, due to their size, while we do not want to split the sequences, to reduce the complexity of the alignment, as this multiplies the intermediate steps, increasing by doing so the risks of errors. It was not the case of *AlignSeqs*, available in the R module called decipher [30]. This latter achieved to perform the MSA in an accurate and rapid way. With this tool, multiple sequence alignments are done by aligning 2 genomes first, and then adds a third genome, etc., until all the sequences are aligned [31].

### Phylogeny

The alignment of multiple genomes of *Mycobacterium* leads to the visualization of synteny blocks, emphasizing the location of large inversions.

A manual reverse of these inversions were possible, leading to an improvement of the alignment of the 65 genomes. This is beneficial for the next stage of the pipeline, namely the phylogenetic investigation. This stage has been performed using RAxML, in which the phylogenetic tree is reconstructed according to a maximum likelihood approach [32]. Note that, thanks to the manual reverse of inversions, the obtained tree has been computed using almost all the complete genomes (only columns with indels are ignored), while without this manual operation, all columns inside the inversion are disregarded. Being based on almost all the genomes, and being strongly supported according to bootstrap values, the obtained tree is trustworthy, and we can reasonably consider it as a backbone to reconstruct ancestral states of MTBC nucleotides.

The proposed ancestral reconstruction is in two parts: 1-length modifications (SNPs and indels) are first considered, before investigating larger modifications (insertion, deletion, or duplication of large scale subsequences). These two case are detailed below.

### Ancestral reconstruction: the mononucleotidic variants case

The treatment is divided in two sub-parts: insertion-deletions on the one hand, and single nucleotide polymorphisms on the other hand. The second case is simple, and its difficulty is only in the separation between real SNPs and polymorphism induced by an indel recombination. The first case is more complicated, as indels may be related to mobile elements or tandem repeats. These two cases are detailed below.

Ancestral reconstruction of SNPs is realized as follows. We first compute the marginal probability distributions in each nucleotide of internal vertices in the phylogeny obtained previously. Assuming a site independence, we have applied the sum-product message passing method [33] to calculate these distributions. This method has been applied by using PHAST [34], which is able to reconstructs ancestral indels too (parsimony approach).

### Ancestral reconstruction: the case of larger variants

In the case of mid-size modifications over time, a string algorithm has been first designed to detect sequence inversions (even in the case of small and noisy ones). However, and due to the fact that MTBC complex is reputed to evolve in a clonal manner, only artifacts have been detected by applying this algorithm on supercomputer facilities. This will not be the case if this pipeline is applied to more recombinating bacteria like the *Pseudomonas* or *Yersinia* genus. Note that, up to now, duplications have not yet been regarded, as the synteny block analysis performed previously has shown that large scale duplications have not occurred in the MTBC case.

**Fig. 1:**
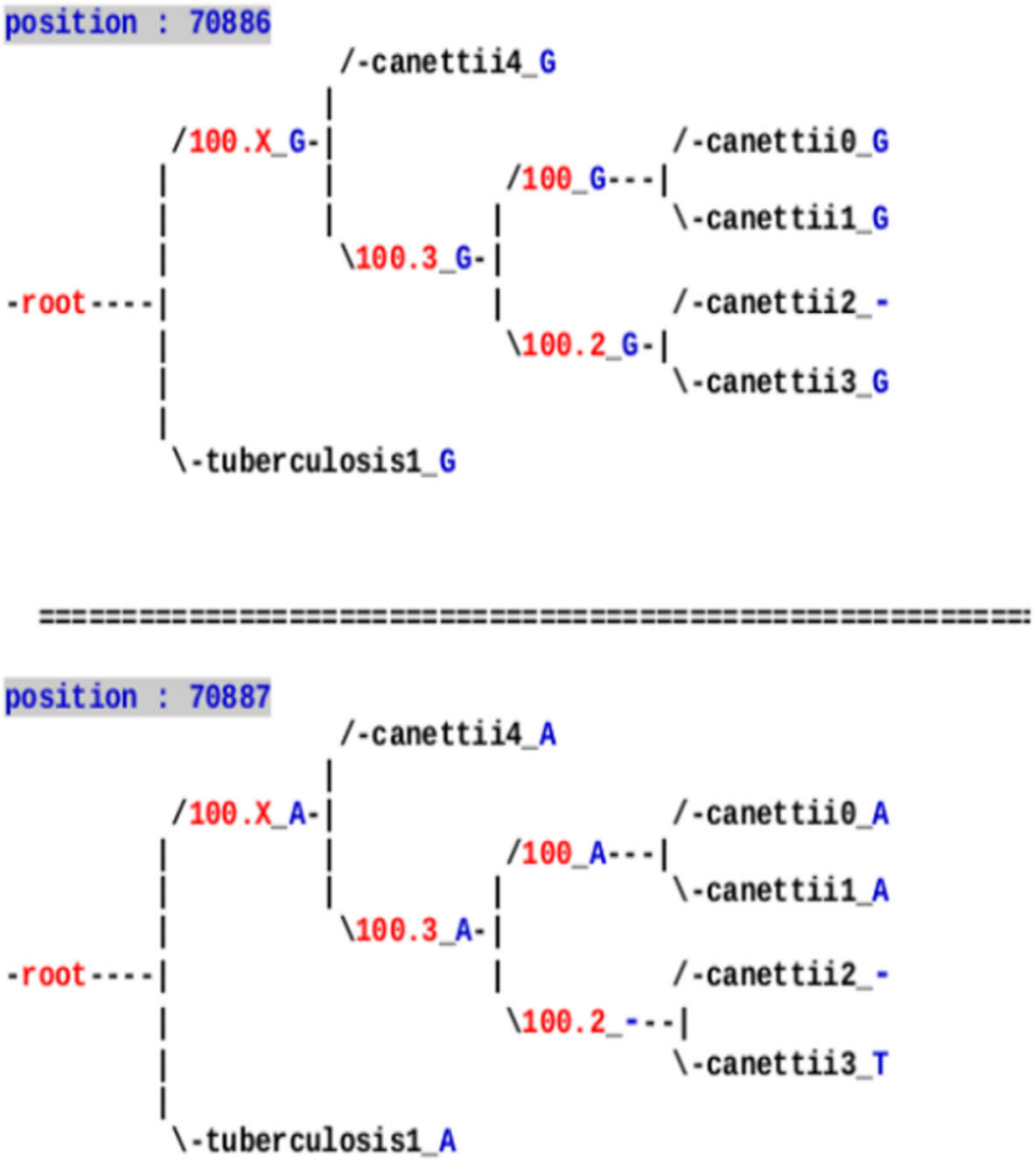
Indels on internal nodes of the tree of some *M. canettii* species

**Fig. 2:**
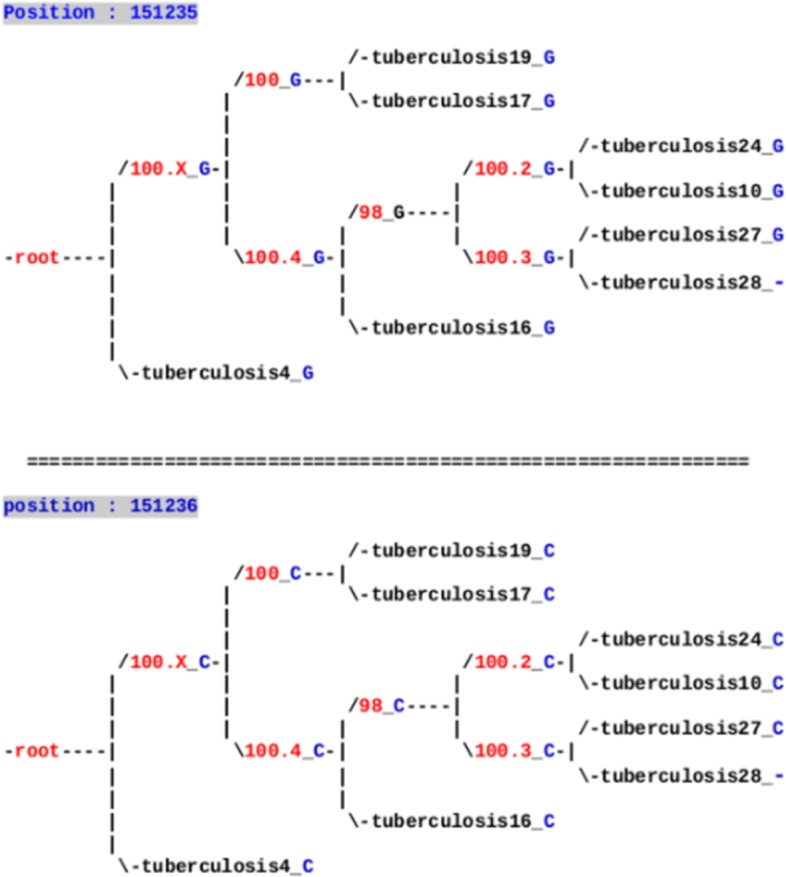
Ancestral reconstruction of one problematic indel in the alignment

Conversely, midsize indels and SNPs have been investigated in details by using PHAST. This investigation has allowed us to notice that: (1) In most of the cases, the situation is obvious, leading either to a deletion or an insertion at a well specific location inside the phylogenetic tree, like in Figs. 1 and 2. (2) These larger variants events are rare in various lineages (e.g., *tuberculosis*), as illustrated in Table 2. (3) In the case of indels of size ≥ 2, the parsimony approach of PHAST produces frequently a wrong ancestral state deduction, which must be modified by hand. Note that its competitors have been tested too, and they all presented worse reconstructions on our specific dataset. (4) The inserted sequence has, in general, not faced additional mutations over times.

**Table 2:** Single nucleotide polymorphism between species (100.X is the name of an ancestral species, cf. the phylogeny)

	*M. canettii SNPs*		*M. tuberculosis SNPs*	
Father	Children	No. of SNPs	Children	No.of SNPs
*100*	*canettii0*	1	*tuberculosis19*	5
	*canettii1*	9	*tuberculosis17*	14
*100.2*	*canettii2*	1041	*tuberculosis24*	1
	*canettii3*	12398	*tuberculosis10*	0
*100.3*	*100*	28	*tuberculosis27*	0
	*100.2*	735	*tuberculosis28*	0
*98*	-	-	*100.2*	1
	-	-	*100.3*	0
100.4	*-*	-	*98*	0
	*-*	-	*tuberculosis16*	1
*100.X*	*100.3*	111	*100*	5
	*canettii4*	438	*100.4*	1

This semi-automatic pipeline for ancestral genomes has finally succeeded to reconstruct the genomes at each internal node of the tree, which can be done because the number of recombination of more than one nucleotide is low. These recombinations have mainly been deduced manually, while state-of-the-art tools have not been able to reach an acceptable level of accuracy.

**Fig. 3:**
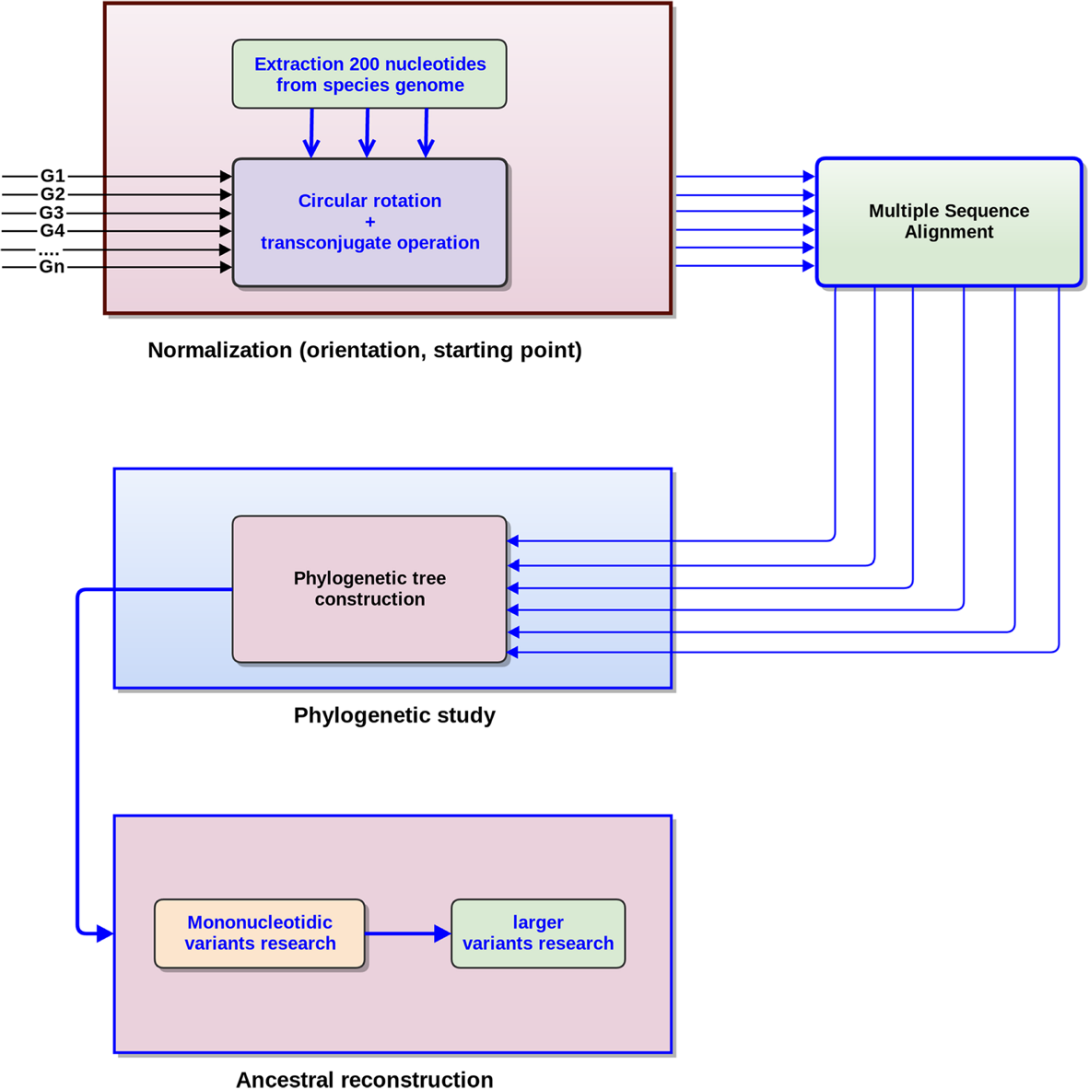
Flowchart of the proposed approach

**Fig. 4:**
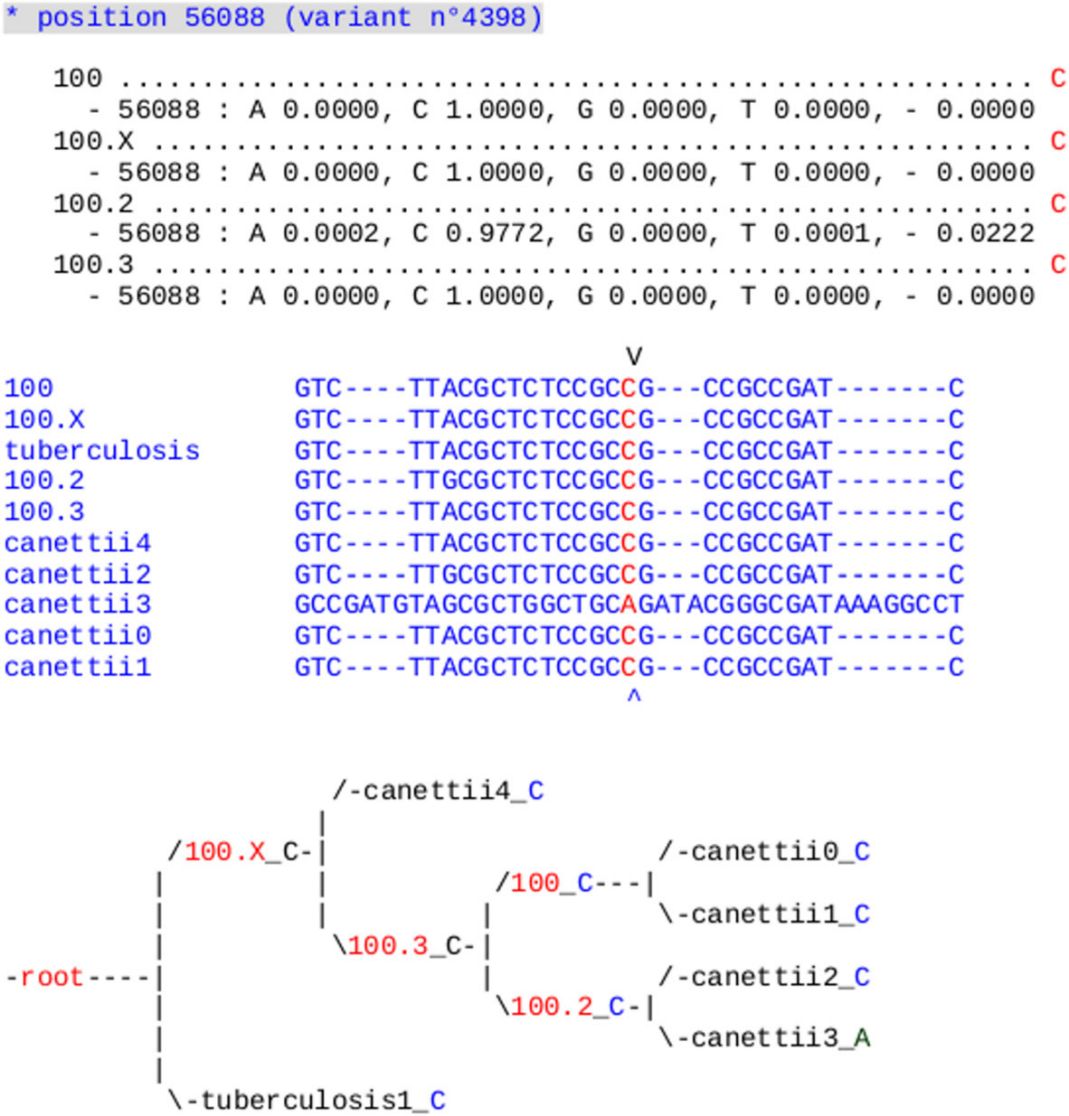
Ancestral reconstruction of a *M. canettii* SNP

Figure 3 summarizes all the ancestral reconstruction process, in which the gray boxes are operated manually, while the other stages are automatic. Indeed, obtained results on mononucleotidic variants have been carefully checked by naked eye, as the number of such variants is lower than one hundred, while ad hoc algorithms were designed to deal with variants of larger size, see Fig. 4.

### CRISPR investigation

Another particular DNA pattern that can evolve through Evolution is the so-called CRISPR one. CRISPR refers to repeated DNA sequences that help to preserve organisms from noticeable threats like viruses. These sequences are a fundamental component of some immune systems, which helps to protect their organism’s health. Such repeated DNA sequences are found in archaeal and bacterial genomes. These sequences range in size from 23 to 47 base pairs.

The name of CRISPR refers to an acronym which stands for Clustered Regularly Interspaced Short Palindromic Repeat [35, 36]. The CRISPR system was initially found as part of an immune system of sorts in some bacteria, used for cutting apart foreign DNA. It consists of two parts of the protein itself, which is the workhorse of the CRISPR system: a bacterial enzyme named Cas9, and a small RNA, called the guide RNA, that matches the DNA sequence to be nicked [37].

## Results

### The Case of Mycobacterium Tuberculosis Complex

**Fig. 5:**
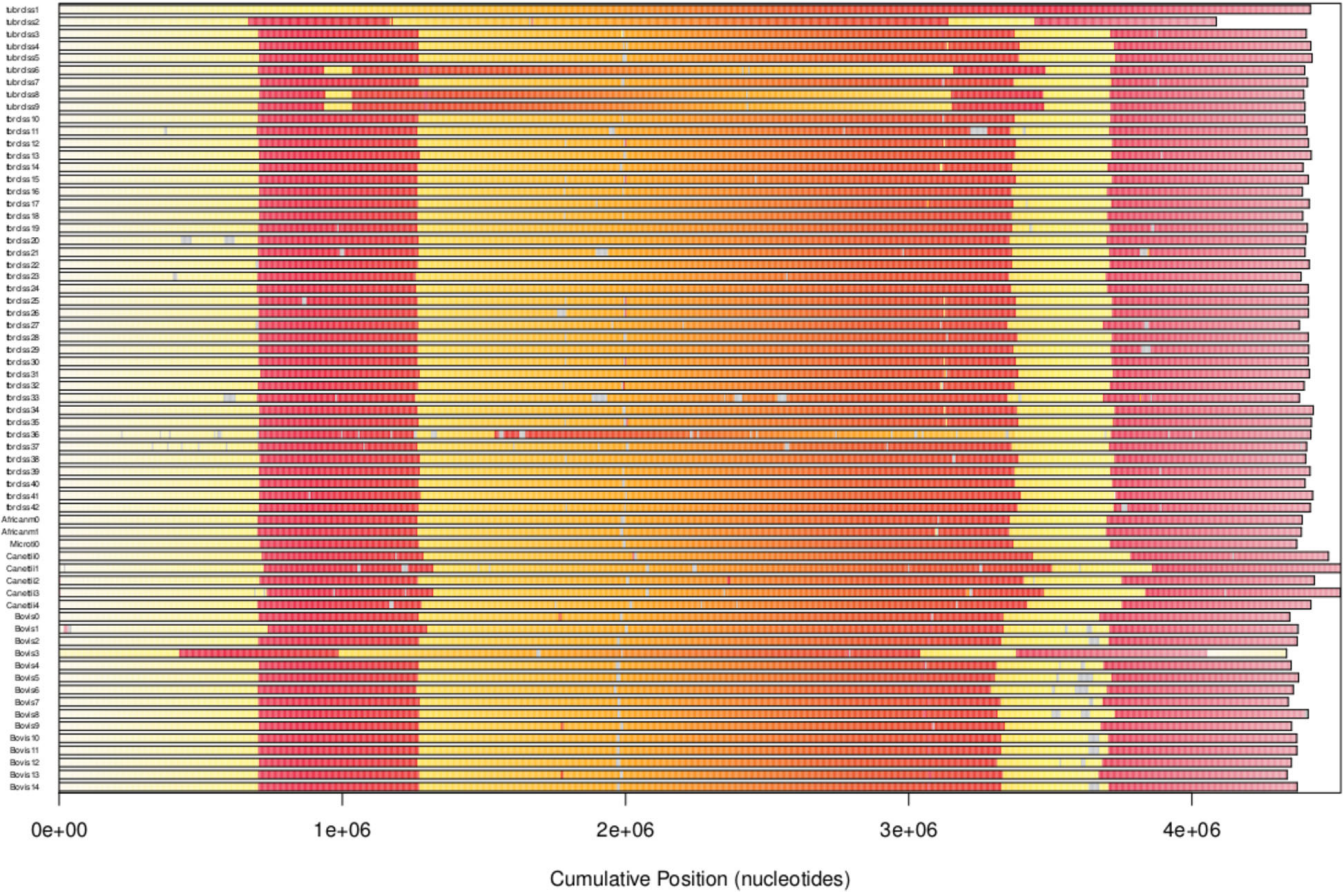
Synteny blocks of *Mycobacterium* strains available online

All the 65 *Mycobacterium* genomes have been aligned thanks to the *AlignSeqs* function described previously. We thus obtained a first representation of synteny of all of them, see Fig. 5. As can be seen, genomes are very similar in the MTBC case, and only a low number of recombinations have occurred within these genomes.

**Fig. 6:**
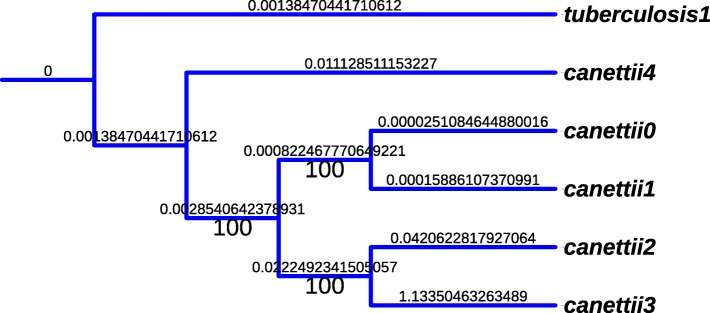
*M. canettii* phylogeny (outgroup: *M. tuberculosis*)

**Fig. 7:**
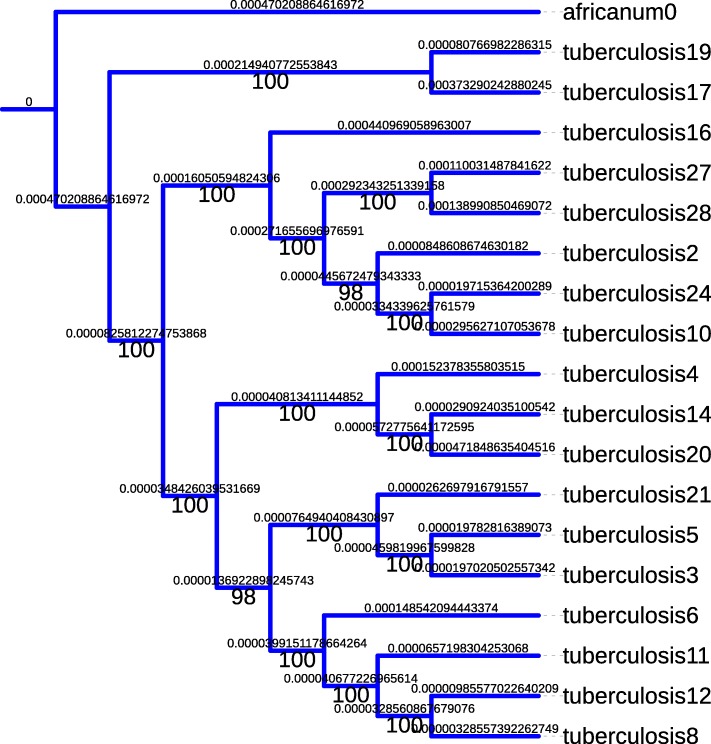
*M. tuberculosis* phylogeny (GTR Gamma model and outgroup:*M. africanum*)

As an illustrative example of the phylogenetic study depicted in Sec. 3, the phylogeny of *M. canettii* is represented in Fig. 6 (outgroup: *M. tuberculosis*). We selected the GTR Gamma model of nucleotide substitution as recommended by JModelTest 2.0, and the tree has been computed by RAxML. Note that the obtained tree is well-supported, as well as in the *M. tuberculosis* cased, whose supports are larger than 98% (cf. Fig. 7). Indeed, with these bacteria, we have not to find the most supported tree based on the largest subset of core genes, as aligning the whole complete genomes leads to a well supported tree: it is not possible to improve the results, which is nice as the core genome is many times greater than in the chloroplast case (and so, it is not sure that the heuristic approach presented in our previous articles [32, 38, 39] can succeed to find the optima).

The obtained results on mononucleotidic variants have been humanly verified, which has been possible due to a low number of variants (cf., for instance, to Tables 3 and 4).

**Table 3:** Number of columns of the MSA with SPNs or indels for *M. canettii* (large deletions are counted character by character)

	*canettii0*	*canettii1*	*canettii2*	*canettii3*	*canettii4*	*tuberculosis1*
***tuberculosis1***	3354	1150	27437	61346	7510	0
***canettii4***	4833	7971	27468	60987	0	7510
***canettii3***	60957	61233	62717	0	60987	61346
***canettii2***	27256	27260	0	62717	27468	27437
***canettii1***	3524	0	27260	61233	7971	1150
***canettii0***	0	3524	27256	60957	4833	3354

Species entries are in boldface

**Table 4:** Variations in the alignment of the *M. tuberculosis* clade under consideration

	*tuberculosis4*	*tuberculosis19*	*tuberculosis17*	*tuberculosis16*	*tuberculosis27*	*tuberculosis28*	*tuberculosis24*	*tuberculosis10*
***tuberculosis4***	0	199770	214401	219205	216387	217235	216919	217186
***tuberculosis19***	199770	0	212403	219039	216908	216672	216726	216953
***tuberculosis17***	214401	212403	0	216808	216534	217011	216786	216882
***tuberculosis16***	219205	219039	216808	0	216669	216916	216251	216678
***tuberculosis27***	216387	216908	216534	216669	0	142974	189148	199505
***tuberculosis28***	217235	216672	217011	216916	142974	0	189460	199412
***tuberculosis24***	216919	216726	216786	216251	189148	189460	0	194315
***tuberculosis10***	217186	216953	216882	216678	199505	199412	194315	0

Species entries are in boldface

**Fig. 8:**
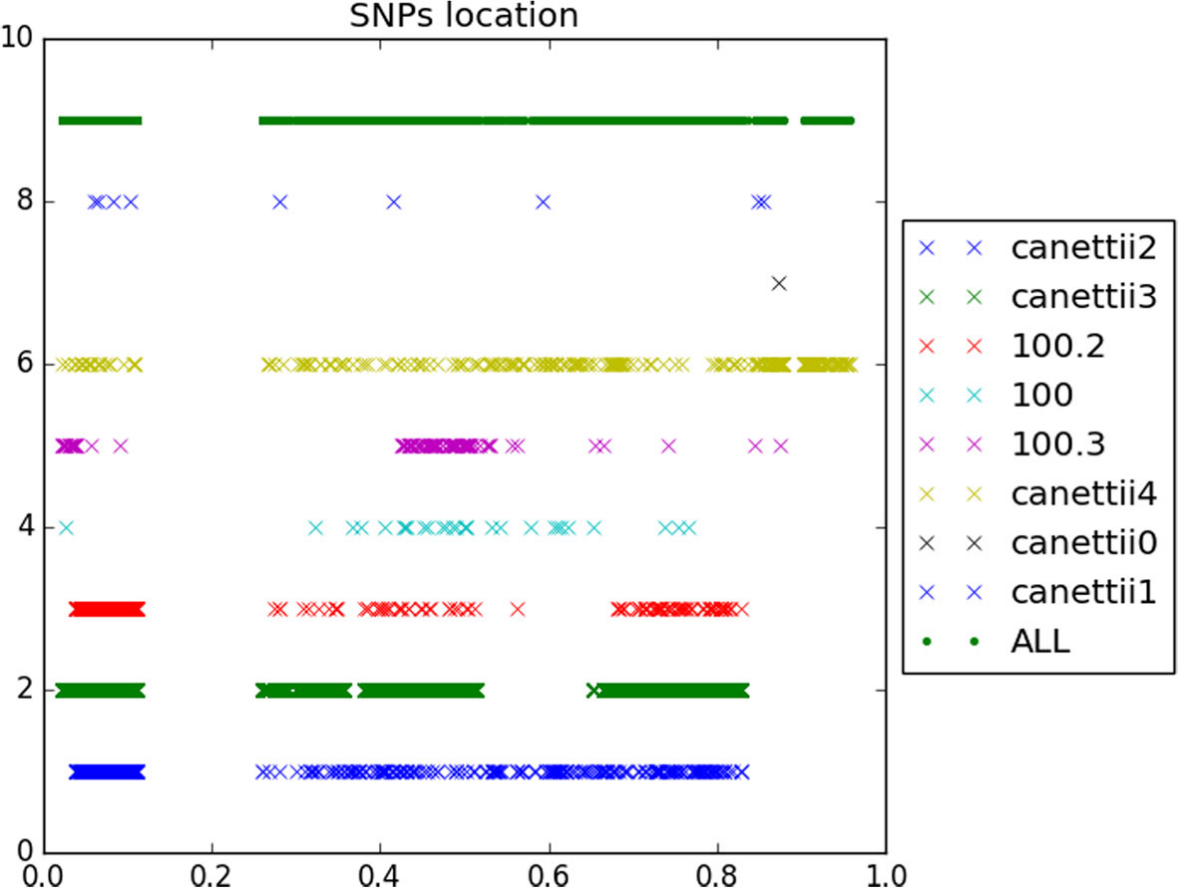
SNPs location of mononucleotidic variants of *M. canettii*

**Fig. 9:**
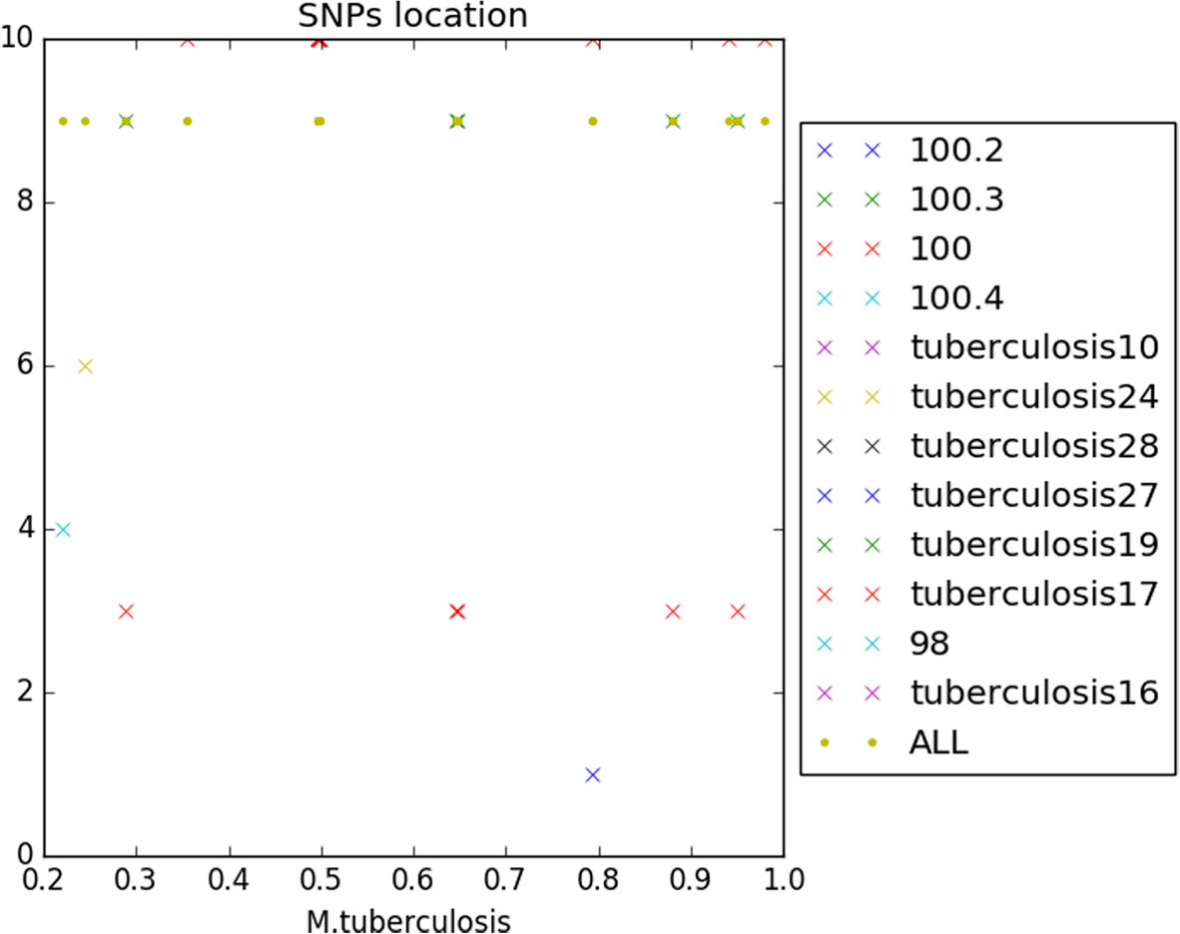
SNPs location of mononucleotidic variants of *M. turberculosis*

166 indels and 2956 SNPs have finally been detected, when considering the 5 *M. canettii* (see Fig. 8). Figure 9, for its part, collects the positions of the 25 indels and 394 SNPs that have been detected in the clade of the 8 *M. tuberculosis*.

**Fig. 10:**
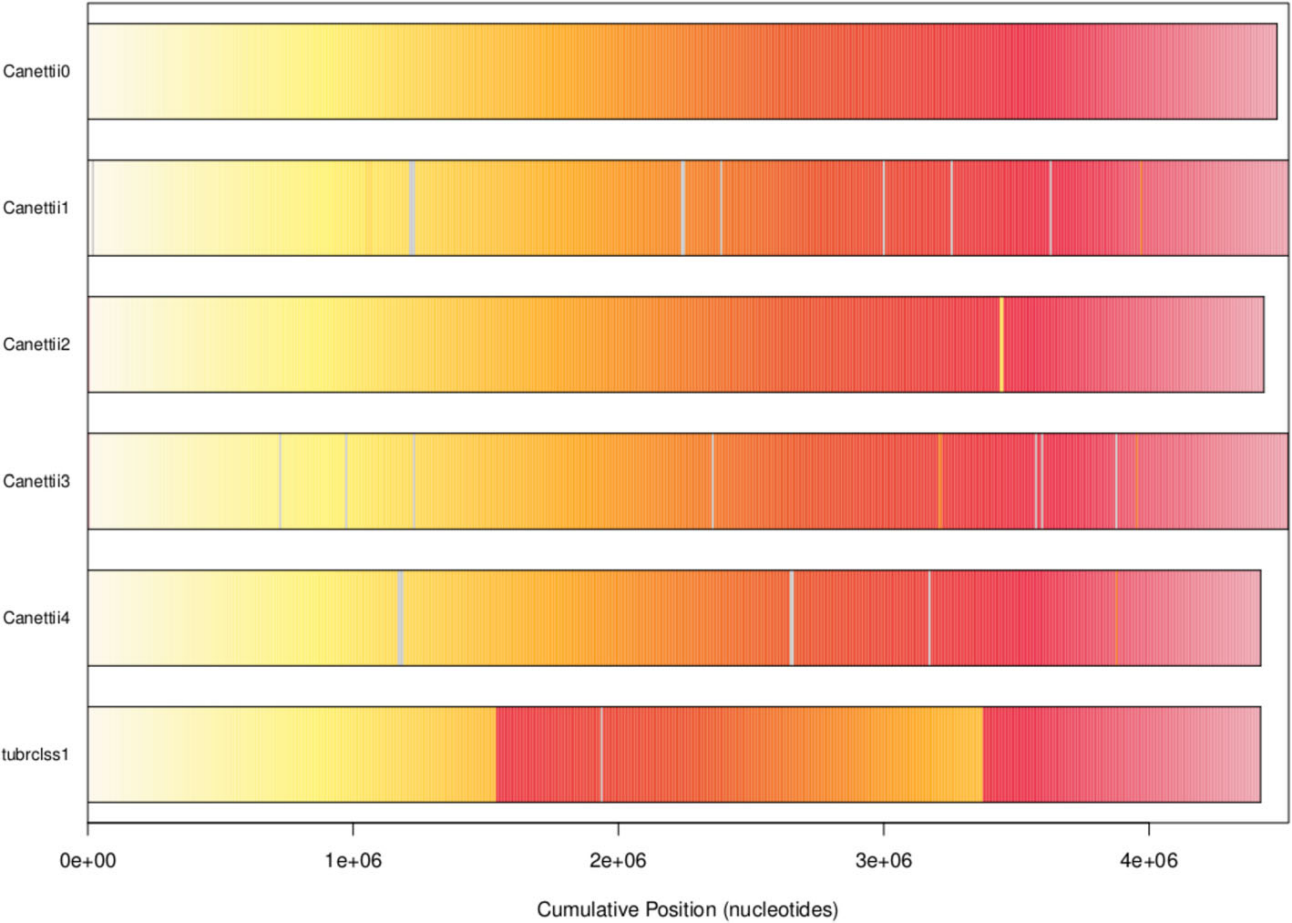
Synteny blocks in *M. canettii*. Each genome is colored according to the position of the corresponding region in the first genome (gray if a region is unshared)

In the considered *Mycobacterium* strains, only a few important inversions have been detected, such as the inversion present in the last ancestor of *140070010*, *CIPT 140010059*, *140070017*, *140060008*, and *140070008*, as shown in Fig. 10. 99% of DNA sequence identity has been obtained when considering all the blocks of synteny of *tuberculosis*. We can conclude that these genomes are highly conserved: highly similar regions without any rearrangement, with only small indels and a large inversion.

We can conclude from this study that ancestral genome reconstruction is possible when considering close or clonal bacteria, and all the material needed in such a pipeline has been designed. But, for the sake of comparison, it may be interesting to deep investigate the results of this semi-automatic reconstruction method on a quite more stable genus, namely the *Brucella*, on which human validation of algorithm results is easier (see Tables 5, 6 and 8 for an illustration of their alignment and SNP differences). Such new investigations are conducted in the next section.

**Table 5:** Differences in the alignment on chromosome 1 of *abortus*

	*abortus0*	*abortus1*	*abortus2*	*abortus3*	*abortus4*	*abortus5*	*abortus6*	*abortus7*	*abortus8*	*abortus9*	*abortus10*	*abortus11*	*abortus12*	*melitensis1*
***abortus0***	0	2320	1030	4304	7194	7481	5308	4891	4850	7837	839	12693	4695	18486
***abortus1***	2320	0	1772	5150	6658	8371	4911	5071	5030	8022	1762	12841	5621	16724
***abortus2***	1030	1772	0	3996	6866	7116	5033	4603	4576	7470	537	12958	4385	18049
***abortus3***	4304	5150	3996	0	10010	5955	2649	853	2462	6271	3800	11488	4738	16568
***abortus4***	7194	6658	6866	10010	0	13161	9784	9884	9892	12820	6601	17617	10413	22727
***abortus5***	7481	8371	7116	5955	13161	0	6834	6408	6441	425	6911	15180	7869	16608
***abortus6***	5308	4911	5033	2649	9784	6834	0	2103	505	6494	4807	11411	5745	16113
***abortus7***	4891	5071	4603	853	9884	6408	2103	0	1907	6055	4393	11534	5321	16337
***abortus8***	4850	5030	4576	2462	9892	6441	505	1907	0	6102	4350	11524	5342	16581
***abortus9***	7837	8022	7470	6271	12820	425	6494	6055	6102	0	7253	14833	8210	16283
***abortus10***	839	1762	537	3800	6601	6911	4807	4393	4350	7253	0	12818	4157	17940
***abortus11***	12693	12841	12958	11488	17617	15180	11411	11534	11524	14833	12818	0	14057	24464
***abortus12***	4695	5621	4385	4738	10413	7869	5745	5321	5342	8210	4157	14057	0	18905
***melitensis1***	18486	16724	18049	16568	22727	16608	16113	16337	16581	16283	17940	24464	18905	0

Species entries are in boldface

**Table 6:** Single nucleotide polymorphism in *Brucella melitensis*

*Chromosome 1* SNPs		
Fathers	Children	No. of SNPs
*100.4*	*100.3*	64
	*melitensis1*	74
*100.2*	*melitensis3*	106
	*melitensis2*	8
*100.X*	*100.5*	4458
	*melitensis0*	104
*100*	*melitensis6*	840
	melitensis5	997
*100.5*	*100*	372
	100.4	689
*100.3*	*100.2*	23
	melitensis7	26

### The Case of *Brucella* genus

The pipeline presented in the previous section is now applied on another genus, namely the *Brucella* one, for the sake of comparison and to broader the discussion. Complete sequences of the 47 available genomes have been downloaded from NCBI, namely by species: *B. abortus* (14 genomes), *melitensis* (8), *sui* (16), *ovis* (1), *canis* (3), *ceti* (2), *pinnipedialis* (2), *neotomae* (0), *microti, inopinata*, and *vulpis*, as described in Table 7.

**Table 7:** *Brucella* genus: genome information

Accession (GenBank)	Organism name	Sequence length(bp)	Nickname
NC_006932.1	*Brucella abortus biovar 1 str. 9-941 chromosome 1*	2,124,241	*abortus0*
NC_006933.1	*Brucella abortus biovar 1 str. 9-941 chromosome 2*	1,162,04	
NC_010742.1	*Brucella abortus S19 chromosome 1*	2,122,487	*abortus1*
NC_010740.1	*Brucella abortus S19 chromosome 2*	1,161,449	
NC_016795.1	*Brucella abortus A13334 chromosome 1*	2,123,773	*abortus2*
NC_016777.1	*Brucella abortus A13334 chromosome 2*	1,162,259	
NZ_CP007663.1	*Brucella abortus strain 63 75 chromosome 1*	2,124,677	*abortus3*
NZ_CP007662.1	*Brucella abortus strain 63 75 chromosome 2*	1,155,633	
NZ_CP007681.1	*Brucella abortus strain BDW chromosome 1*	2,128,683	*abortus4*
NZ_CP007680.1	*Brucella abortus strain BDW chromosome 2*	1,160,817	
NZ_CP007682.1	*Brucella abortus strain BER chromosome 1*	2,125,180	*abortus5*
NZ_CP007683.1	*Brucella abortus strain BER chromosome 2*	1,163,338	
NZ_CP007700.1	*Brucella abortus strain NCTC 10505 chromosome 1*	2,123,620	*abortus6*
NZ_CP007701.1	*Brucella abortus strain NCTC 10505 chromosome 2*	1,161,669	
NZ_CP007705.1	*Brucella abortus bv. 9 str. C68 chromosome 1*	2,124,100	*abortus7*
NZ_CP007706.1	*Brucella abortus bv. 9 str. C68 chromosome 2*	1,155,846	
NZ_CP007709.1	*Brucella abortus bv. 6 str. 870 chromosome 1*	2,124,096	*abortus8*
NZ_CP007710.1	*Brucella abortus bv. 6 str. 870 chromosome 2*	1,157,058	
NZ_CP007738.1	*Brucella abortus strain BFY chromosome 1*	2,124,832	*abortus9*
NZ_CP007737.1	*Brucella abortus strain BFY chromosome 2*	1,1633,26	
NZ_CP007765.1	*Brucella abortus bv. 2 str. 86/8/59 chromosome 1*	2,123,991	*abortus10*
NZ_CP007764.1	*Brucella abortus bv. 2 str. 86/8/59 chromosome 2*	1,162,137	
NZ_CP008774.1	*Brucella abortus strain BAB8416 chromosome 1*	2,116990	*abortus11*
NZ_CP008775.1	*Brucella abortus strain BAB8416 chromosome 2*	1,156,120	
NZ_CP009626.1	*Brucella abortus 104M chromosome 2*	1,162,580	*abortus12*
NZ_CP009625.1	*Brucella abortus 104M chromosome 1*	2,122,847	
NZ_LN997863.1	*Brucella sp. F60 genome assembly BVF60 chromosome 1*	2,177,010	*sp*
NZ_LN997864.1	*Brucella sp. F60 genome assembly BVF60 chromosome 2*	1,061,127	
NZ_CP007759.1	*Brucella canis strain RM6/66 chromosome 2*	1,206,801	*canis3*
NZ_CP007758.1	*Brucella canis strain RM6/66 chromosome 1*	2,105,950	
NC_010103.1	*Brucella canis ATCC 23365 chromosome 1*	2,105,69	*canis0*
NC_010104.1	*Brucella canis ATCC 23365 chromosome 2*	1,206,800	
NC_016778.1	*Brucella canis HSK A52141 chromosome 1*	2,107,023	*canis1*
NC_016796.1	*Brucella canis HSK A52141 chromosome 2*	1,170,489	
NZ_CP007629.1	*Brucella canis strain SVA13 chromosome 1*	2,106,955	*canis2*
NZ_CP007630.1	*Brucella canis strain SVA13 chromosome 2*	1,203,360	
NC_022905.1	*Brucella ceti TE10759-12 chromosome 1*	2,117,718	*ceti*
NC_022906.1	*Brucella ceti TE10759-12 chromosome 2*	1,160,316	
NC_007618.1	*Brucella melitensis biovar Abortus 2308 chromosome 1*	2,121,359	*melitensis0*
NC_007624.1	*Brucella melitensis biovar Abortus 2308 chromosome 2*	1,156,948	
NZ_CP008751.1	*Brucella melitensis strain 20236 chromosome 2*	1,185,741	*melitensis7*
NZ_CP008750.1	*Brucella melitensis strain 20236 chromosome 1*	2,126,134	
NZ_CP007762.1	*Brucella melitensis bv. 1 str. 16M chromosome 2*	1,177,791	*melitensis6*
NZ_CP007763.1	*Brucella melitensis bv. 1 str. 16M chromosome 1*	2,116,984	
NZ_CP007761.1	*Brucella melitensis bv. 3 str. Ether chromosome 2*	1,187,961	*melitensis5*
NZ_CP007760.1	*Brucella melitensis bv. 3 str. Ether chromosome 1*	2,122,766	
NC_017283.1	*Brucella melitensis NI chromosome 2*	1,176,758	*melitensis4*
NC_017248.1	*Brucella melitensis NI chromosome 1*	2,117,717	
NC_017247.1	*Brucella melitensis M5-90 chromosome 2*	1,185,778	*melitensis3*
NC_017246.1	*Brucella melitensis M5-90 chromosome 1*	2,126,451	
NC_017245.1	*Brucella melitensis M28 chromosome 2*	1,185 615	*melitensis2*
NC_017244.1	*Brucella melitensis M28 chromosome 1*	2,126,133	
NC_012442.1	*Brucella melitensis ATCC 23457 chromosome 2*	1,185,518	*melitensis1*
NC_012441.1	*Brucella melitensis ATCC 23457 chromosome 1*	2,125,701	
NC_013119.1	*Brucella microti CCM 4915 chromosome 1*	2,117,050	*microti*
NC_013118.1	*Brucella microti CCM 4915 chromosome 2*	1,220,319	
NC_009505.1	*Brucella ovis ATCC 25840 chromosome 1*	2,111,370	*ovis*
NC_009504.1	*Brucella ovis ATCC 25840 chromosome 2*	1,164,220	
NC_015857.1	*Brucella pinnipedialis B2/94 chromosome 1*	2,138,342	*pinnipedialis0*
NC_015858.1	*Brucella pinnipedialis B2/94 chromosome 2*	1,260,926	
NZ_CP007743.1	*Brucella pinnipedialis strain 6/566 chromosome 1*	2,139,033	*pinnipedialis1*
NZ_CP007742.1	*Brucella pinnipedialis strain 6/566 chromosome 2*	1,191,996	
NZ_CP010851.1	*Brucella suis strain Human/AR/US/1981 chromosome 2*	1,207,241	*suis0*
NZ_CP010850.1	*Brucella suis strain Human/AR/US/1981 chromosome 1*	2,107,845	
CP009095.1	*Brucella suis strain ZW043 chromosome 2*	1,215,956	*suis1*
CP009094.1	*Brucella suis strain ZW043 chromosome 1*	2,224,908	
CP009097.1	*Brucella suis strain ZW046 chromosome 2*	1,311,857	*suis2*
CP009096.1	*Brucella suis strain ZW046 chromosome 1*	2,181,422	
NZ_CP008756.1	*Brucella suis strain BSP chromosome 2*	1,410,995	*suis3*
NZ_CP008757.1	*Brucella suis strain BSP chromosome 1*	1,902,870	
NZ_CP007718.1	*Brucella suis bv. 3 str. 686 chromosome 2*	1,190,208	*suis4*
NZ_CP007719.1	*Brucella suis bv. 3 str. 686 chromosome 1*	2,107,052	
NZ_CP007716.1	*Brucella suis strain 513UK chromosome 2*	1,187,980	*suis5*
NZ_CP007717.1	*Brucella suis strain 513UK chromosome 1*	2,131,717	
NZ_CP007696.1	*Brucella suis bv. 2 strain Bs143CITA chromosome 2*	1,398,244	*suis6*
NZ_CP007695.1	*Brucella suis bv. 2 strain Bs143CITA chromosome 1*	1,926,295	
NZ_CP007721.1	*Brucella suis bv. 2 strain Bs396CITA chromosome 2*	1,401,375	*suis7*
NZ_CP007720.1	*Brucella suis bv. 2 strain Bs396CITA chromosome 1*	1,927,083	
NZ_CP007698.1	*Brucella suis bv. 2 strain Bs364CITA chromosome 2*	1,401,378	*suis8*
NZ_CP007697.1	*Brucella suis bv. 2 strain Bs364CITA chromosome 1*	1,927,594	
NC_004310.3	*Brucella suis 1330 chromosome 1*	2,107,794	*suis9*
NC_004311.2	*Brucella suis 1330 chromosome 2*	1,207,381	
NC_010169.1	*Brucella suis ATCC 23445 chromosome 1*	1,923,763	*suis10*
NC_010167.1	*Brucella suis ATCC 23445 chromosome 2*	1,400,844	
NC_017251.1	*Brucella suis 1330 chromosome 1*	2,107,783	*suis11*
NC_017250.1	*Brucella suis 1330 chromosome 2*	1,207,380	
NC_016797.1	*Brucella suis VBI22 chromosome 1*	2,108,637	*suis12*
NC_016775.1	*Brucella suis VBI22 chromosome 2*	1,207,451	
NZ_CP006961.1	*Brucella suis bv. 1 str. S2 chromosome 1*	2,107,842	*suis13*
NZ_CP006962.1	*Brucella suis bv. 1 str. S2 chromosome 2*	1,207,433	
NZ_CP007691.1	*Brucella suis bv. 2 strain PT09143 chromosome 1*	1,926,480	*suis14*
NZ_CP007692.1	*Brucella suis bv. 2 strain PT09143 chromosome 2*	1,398,285	
NZ_CP007693.1	*Brucella suis bv. 2 strain PT09172 chromosome 1*	1,926,716	*suis15*
NZ_CP007694.1	*Brucella suis bv. 2 strain PT09172 chromosome 2*	1,398,326

**Table 8:** Single nucleotide polymorphism in *Brucella abortus*

	*Chromosome 1 SNPs*		*Chromosome 2 SNPs*	
Fathers	Children	No. of SNPs	Children	No. of SNPs
*100.2*	*abortus10*	55	*abortus10*	41
	*abortus0*	72	*abortus0*	38
*100*	*abortus2*	37	*abortus2*	25
	*abortus1*	55	*abortus1*	15
*100.3*	*100*	37	*100*	17
	*100.2*	5	*100.2*	0
*100.X*	100.3	24	100.3	15
	abortus4	84	abortus4	51

Note that the genome of *Brucella abortus* has two circular chromosomes. The first one is 2,124,241 bp long in the *Brucella abortus biovar 1 str. 9-941* reference genome, while the second chromosome is of 1,162,204 bp. Other species in the *Brucella* genus are comparable in genome size. For instance, the *Brucella melitensis strain 16M* is constituted of 3,294,931 bp disseminated in two circular chromosomes: chr. I has 2,117,144 bp, while chromosome II has 1,177,787 bp. On both of these chromosomes, approximately 3100 ORFs were predicted. In the latter, genes encoding for DNA replication, protein synthesis, core metabolism, and cell-wall biosynthesis can be found on both chromosomes [40, 41].

**Fig. 11:**
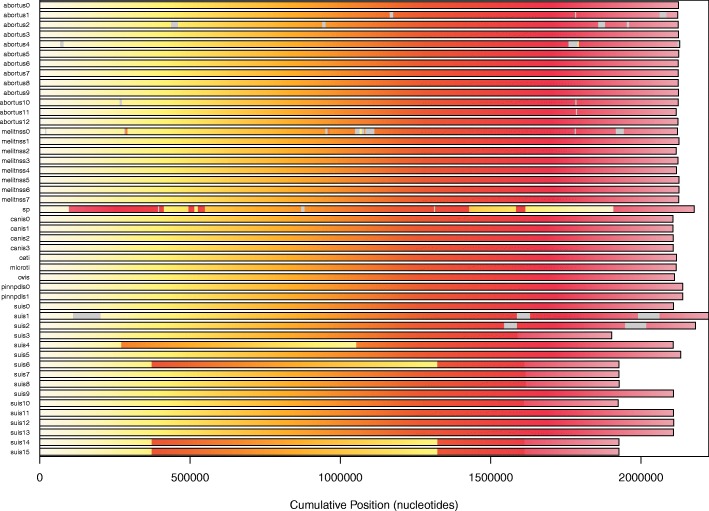
*Brucella*, chromosome 1: a high sequence similarity with little recombination events

**Fig. 12:**
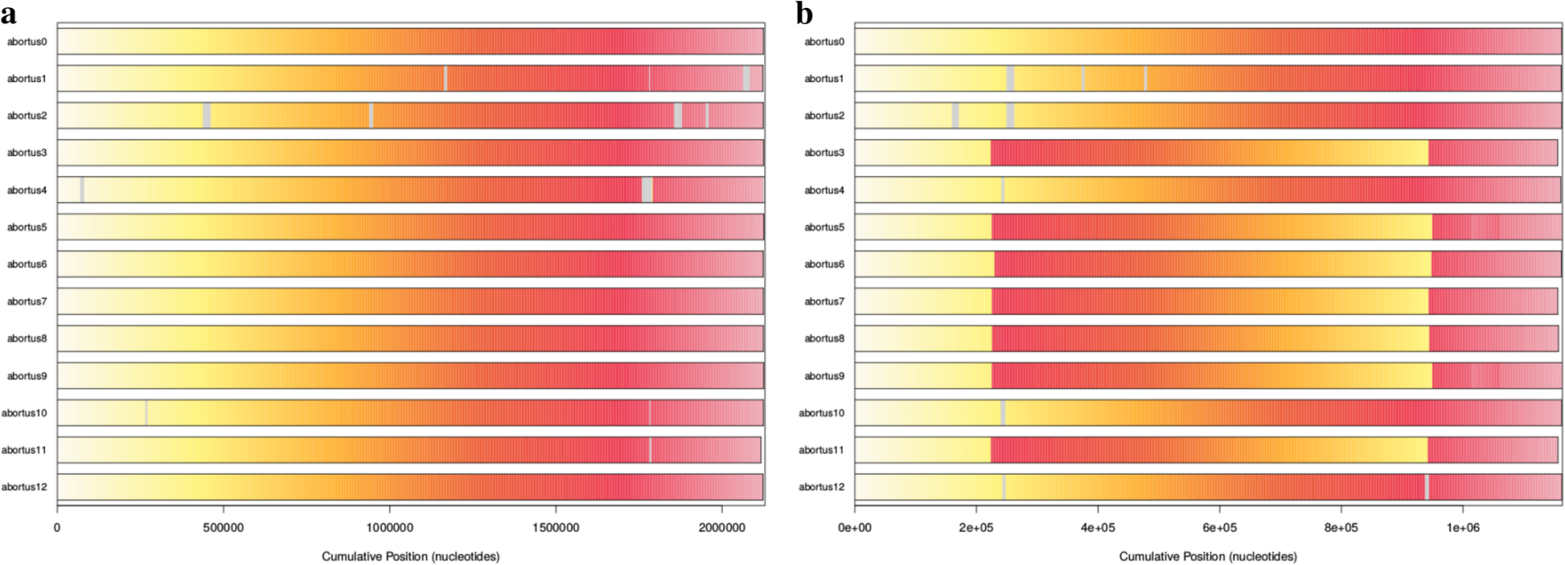
Synteny map of *Brucella abortus* (**a**) chromosome 1 and (**b**) chromosome 2. Genomes investigation tends to show a high sequence similarity with little recombination events. Each genome is colored according to the position of the corresponding region in the first genome, or gray if a region is unshared

We operated the sequences so that they share the same orientation (which may need a transconjugate operation) and the same sequence of 200 nucleotides as starting point (which may require a circular shift), if we except local SNPs. This has been achieved using a local blast, with the beginning of *Brucella abortus* 2308 as an arbitrary reference. After such operations, a syntheny representation of *Brucella* genomes can be obtained, as shown in Fig. 11. The particular case of *B. abortus* is depicted in Fig. 12.

**Fig. 13:**
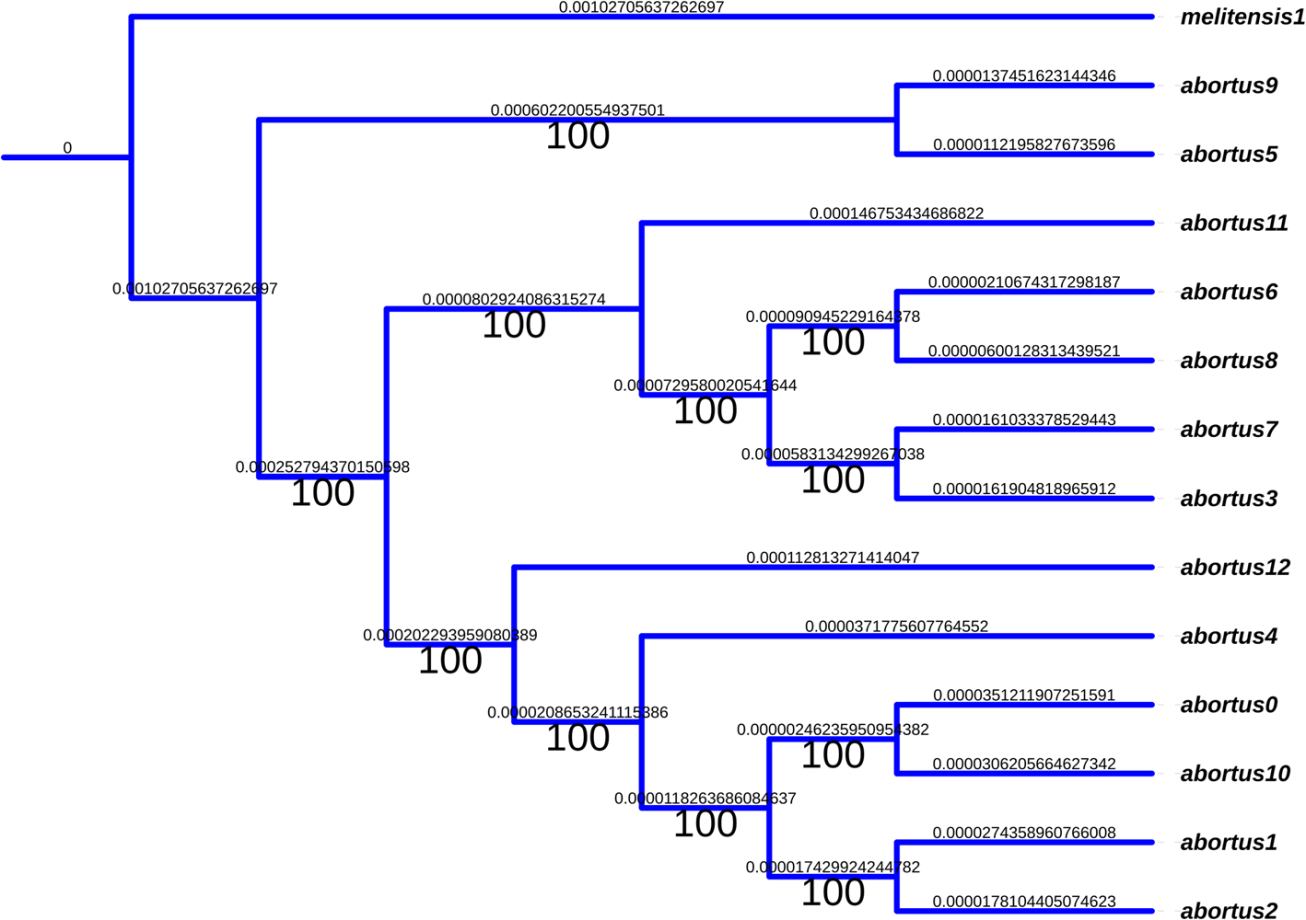
Well-supported phylogeny of *Brucella abortus* species calculated on the entire chromosome 1. The outgroup is *melitensis*, while RaxML has been launched with the GTR Gamma model

**Fig. 14:**
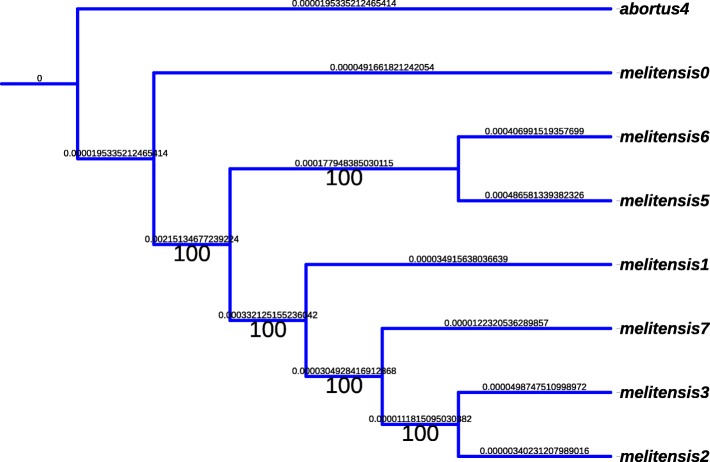
Well supported phylogeny of *Brucella melitensis* species

A few inversions have appeared in this representation. For instance, in the *B.abortus* case, we found a significant inversion at the last common ancestor of strains*“biovar_1_str._9-941”, S19, A13334, “strain_BDW”, “bv._2_str._86/8/59”*, and 104M. We have manually reversed these inversions, so that an accurate alignment of the whole genomes can be performed. Using this alignment, a very well supported phylogenetic tree has been obtained. For the sake of illustration, a subtree corresponding to the phylogeny of the *Brucella abortus* species is depicted in Fig. 13, and in Fig. 14 for *B. melitensis*. It has been obtained using the entire genome sequences with RaxML, GTR Gamma model, and *Brucella melitensis* as outgroup. As can be shown, all branches exhibit a 100% bootstrap support value.

**Fig. 15:**
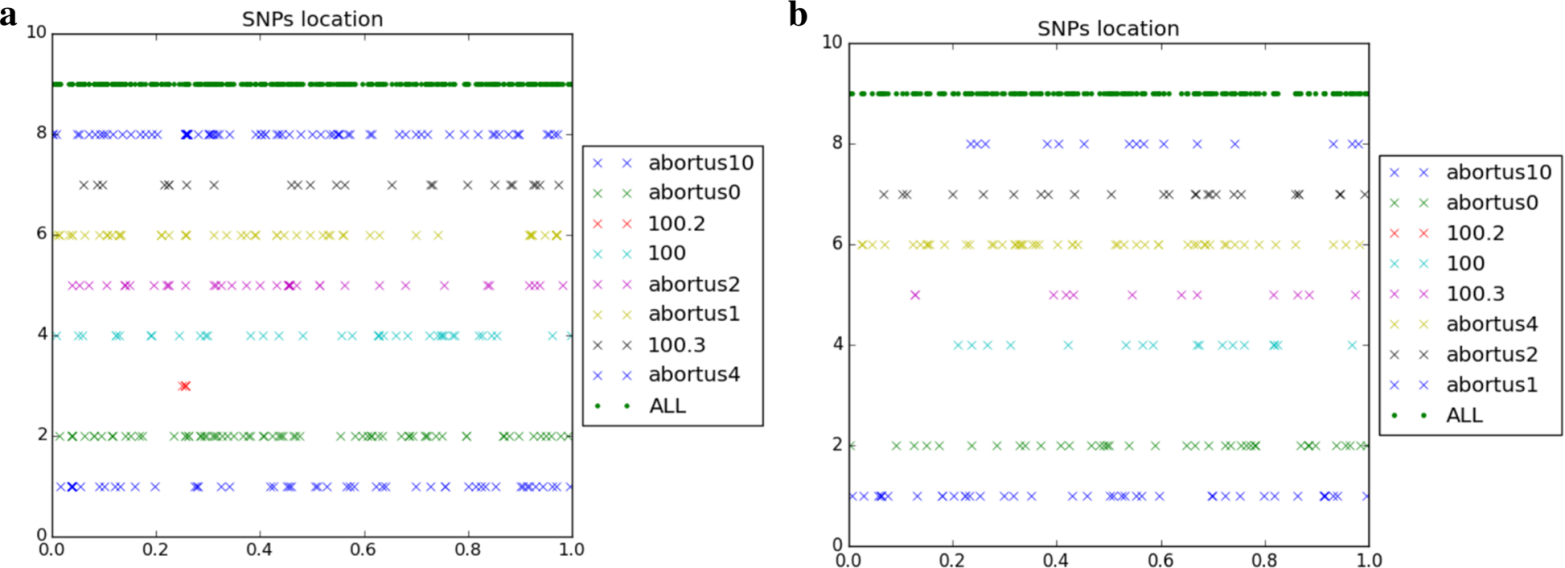
SNPs location in *Brucella abortus* species. (**a**) Chromosome 1, (**b**) chromosome 2

**Fig. 16:**
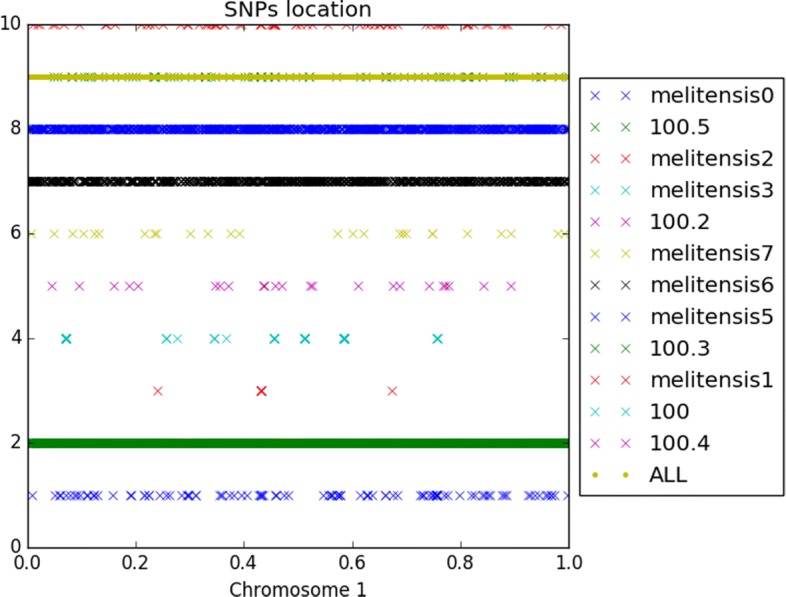
Single nucleotide polymorphism in *Brucella melitensis* species

At this stage, all the material required to attack the ancestral reconstruction of *Brucella* genomes are on hand. We first have focused on the *abortus* and *metilensis* reference species, to investigate the potential origin and the history of the global spread of these *Brucellas*. We have considered the global alignment of both chromosomes 1 and 2 of the available complete strains, using decipher R package [42], and the tree depicted in Figs. 13 and 14. We firstly achieved a comparative whole-genome single nucleotide polymorphism analysis of these strains collected and downloaded from the NCBI. 32 indels and 373 SNPs have been detected in the clade containing these 6 variants of chromosome 2, and 609 SNPs and 325 indels in chromosomes 1, as shown in Fig. 15. The same has been computed for *B. melitensis*, leading to 6178 variants and 335 indels, see Fig. 16. This has been achieved using homemade python scripts on aligned sequences.

**Fig. 17:**
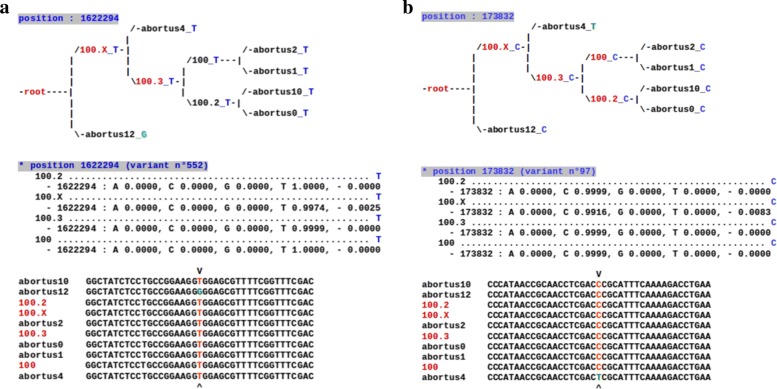
Nucleotides in the ancestral nodes and their children on *Brucella abortus* species. **a** Chromosome 1 **b** chromosome 2

At mononucleotidic variant level, the treatments of SNPs and of indels have been separated. Examples of mononucleotidic ancestral reconstructions are provided in Fig. 17. Differences between ancestors and their children are, for their part, provided in Tables 6 (*abortus*) and 8 (*melitensis*).

**Fig. 18:**
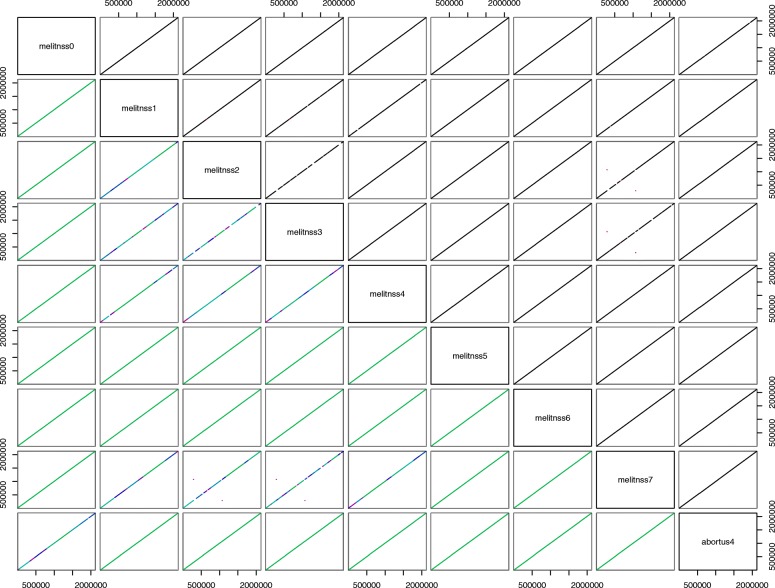
Dotplot of *Brucella melitensis* species, chromosome 1

Figure 12 shows homologous regions among many *Brucella abortus* genomes, as identified by FindSynteny (R). On the one hand, the similarity and preservation of synteny blocks on *Brucella abortus* are especially pronounced in chromosome 1, with highly similar regions and without rearrangement of homologous backbone sequences as shown in Fig. 12a. Chromosome 2, on the other hand, is more diverse. There is above all a significant reversal in the *Brucella abortus* genomes of the clade consisting of abortus 0, 1, 2, 4, 10, and 12 as shown in Fig. 12b. The same information is provided for *B. melitensis* (chromosome 1) in Fig. 18. These differences most likely represent distinct evolutionary origins, for instance related to the nature of functional genes in the two chromosomes.

**Fig. 19:**
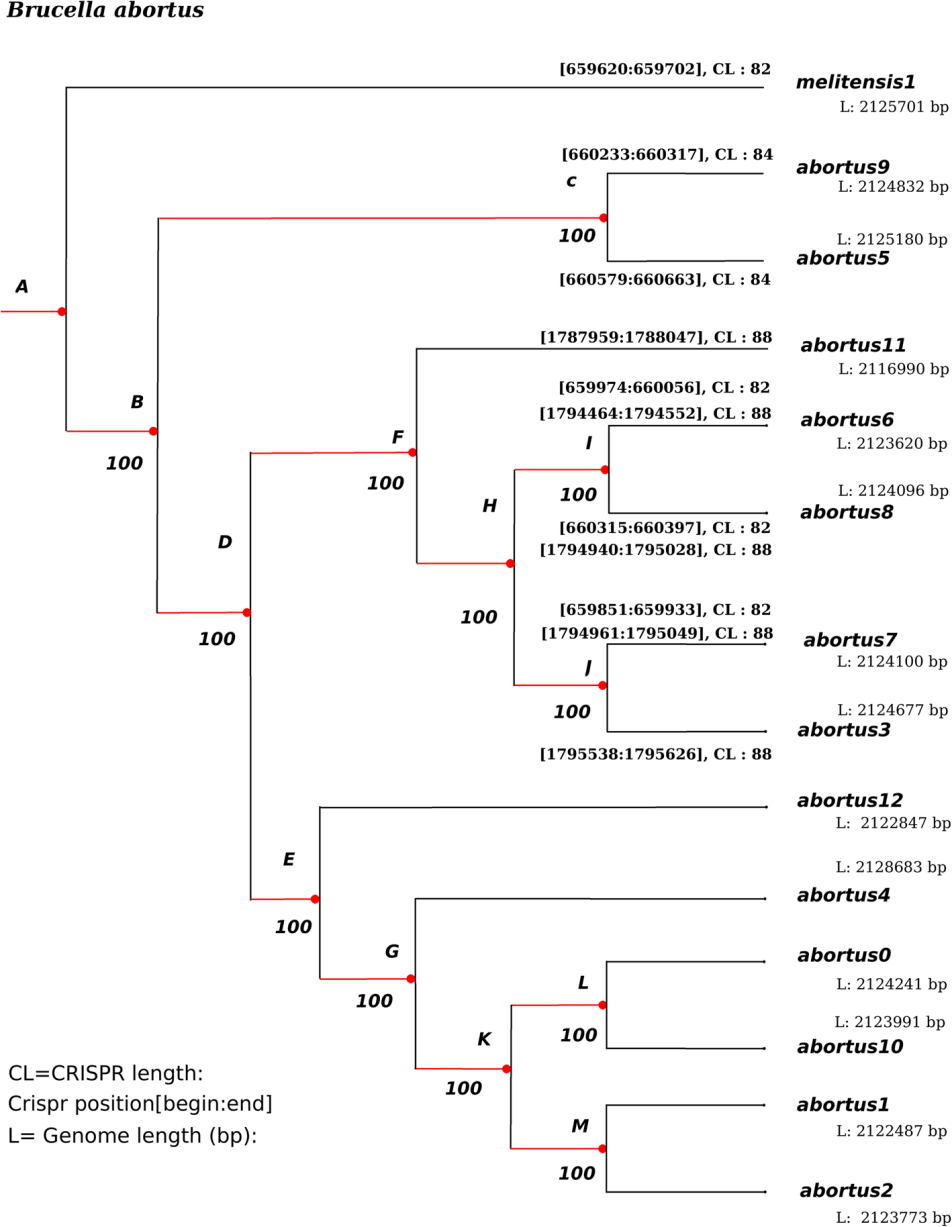
*Brucella abortus* phylogenetic tree: estimation of the CRISPRs length and locations by using the CRISPRFinder web server [36]

**Fig. 20:**
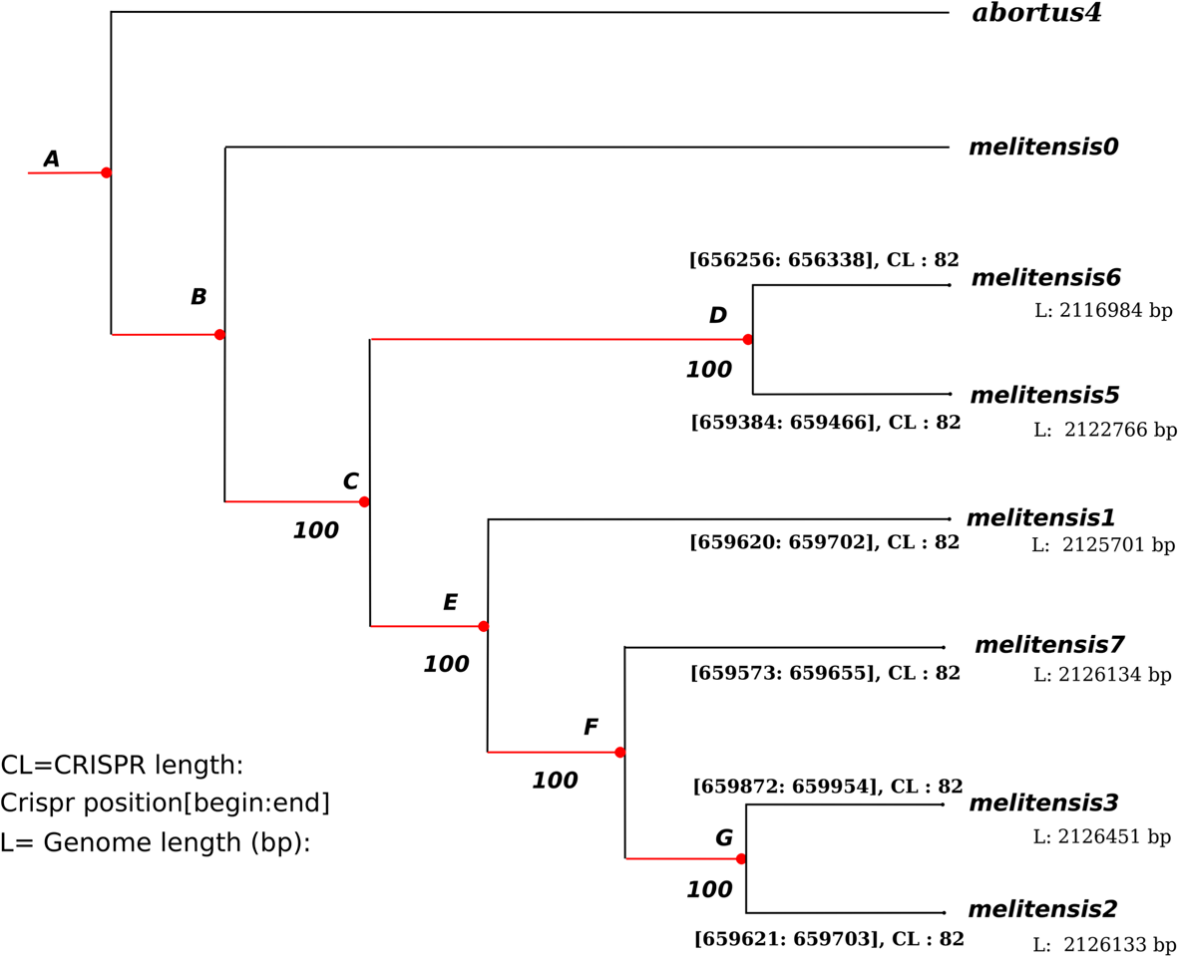
CRISPR investigation in *B. melitensis*

We finally analyzed the CRISPR locus sequences of 14 *Brucella abortus* strains by using CRISPRs web service (http://crispr.i2bc.paris-saclay.fr). The orthologous sequence shared between *Brucella abortus* genomes and the CRISPR spacer have shown a significant similarity of the spacer sequences. Figure 19, for its part, shows the CRISPR space sequence lengths and their positions inside *abortus* genomes. For the *B. melitensis* case, information are provided in Fig. 20.

## Discussion

Various algorithms and methods can be found in the literature to resolve, at least partially, the ancestral genome reconstruction problem. We have shown that these existing methods are not accurate and mature enough to be applied on a real case scenario. This is particularly evident when indels or single nucleotide polymorphisms are mixed with repeated sequences. The main drawback of these methods is that they intend to solve all the cases, while some situations are up-to-now too difficult to be resolved automatically. However, in mid-size genomes that have faced a low number of recombinations over time, as for *Brucella* and *Mycobacterium*, these problematic situations can be signaled, and a human cross-validation can reinforce the accuracy of the ancestral reconstruction algorithm.

As a proof of concept, all ancestral genomes of all *M. canettii* available on the NCBI database have been reconstructed, as well as all the ancestors of the available *M. tuberculosis* complete genomes. At each time, the single nucleotide polymorphism level has first been investigated, before considering the cases of indels and large scale recombination.

Obtained results show that a concrete and accurate reconstruction can be achieved by coupling human decisions on problematic situations with automatic inference of ancestral states in easy to resolve ones, at least for some non recombinant bacteria. With such a reconstruction, it may be possible to deeply investigate the evolution of genomes over time, and possibly to predict their future modifications.

## Conclusion

In this article, we presented a semi-automatic pipeline that achieves to completely and accurately reconstruct the ancestral genomes of some clonal bacteria. In this pipeline, the case of SNPs and indels of 1 nucleotide has been resolved using the sum-product message passing algorithm, while larger modifications have been studied by a parsimony approach coupled with a manual deduction.

The obtained ancestors have not yet been investigated in this study, as it was not the objective of this proof of concept. They will be studied with ad hoc algorithms to design, to investigate the evolution of gene content on the one hand, and of mobile elements on the other hand [43, 44]. The rate at which such loss or gain occurs will be examined carefully, and we will study if some particular functionality are more affected by these mutations. To say this differently, we will investigate if modifications have a real impact during the evolution of genomes.

## Abbreviations

Indels: Insertion or deletion of bases in the genome of an organism
MTB: *Mycobacterium tuberculosis*
MTBC: *Mycobacterium tuberculosis complex*
SNPs: Single nucleotide polymorphisms
TB: *Tuberculosis*
WHO: World health organization
